# Insights into Population Status and Habitat Patches of Conservation Concern for the Endangered Indian Pangolin (*Manis crassicaudata*) in Nowshera District, Northwestern Pakistan

**DOI:** 10.3390/biology13090727

**Published:** 2024-09-16

**Authors:** Romaan Hayat Khattak, Shakeel Ahmad, Tahir Mehmood, Hongliang Dou, Haiyang Gao, Song Sun, Yan Hua

**Affiliations:** 1 Guangdong Provincial Key Laboratory of Silviculture, Protection and Utilization, Guangdong Academy of Forestry, Guangzhou 510520, China; romaanhayatkhattak@nefu.edu.cn (R.H.K.); douhl@sinogaf.cn (H.D.); gaohy@sinogaf.cn (H.G.); ecologysun@163.com (S.S.); 2 Carnivore Conservation Laboratory, Department of Zoology, Quaid-I-Azam University, Islamabad 45302, Pakistan; ahmadsa.ccl@gmail.com; 3 School of Natural Sciences (SNS), National University of Sciences and Technology (NUST), Islamabad 44000, Pakistan; tahime@gmail.com

**Keywords:** species distribution modeling, sign survey, living burrows, Nizampur National Park, *Manis crassicaudata*, threatened species

## Abstract

**Simple Summary:**

The Indian pangolin, an endangered species found in Pakistan, was studied in the Nowshera district to assess its population and suitable habitat. We used the line transect method and recorded 56 signs of pangolins, including burrows and digging, over a 156 km^2^ area. The estimated population was very low, with only 29 individuals and a density of 0.013 individuals/km^2^. The MaxEnt model, which predicts habitat suitability, showed that 12.01% of the area was highly suitable and 13.61% was moderately suitable for the pangolin. To protect this fragile population and available habitat patches, the results obtained in the current study recommend stronger law enforcement and conservation efforts.

**Abstract:**

The Indian pangolin (*Manis crassicaudata*) stands out among the four surviving species of Asian pangolins, being the sole species present in Pakistan and listed as endangered owing to trafficking and illicit commerce. In the present study, we explored the population status of the Indian pangolin and the existing suitable habitats in Nowshera district, Pakistan. We employed the line transect method to confirm the species presence and subsequent population estimation. In a survey effort of 156 km^2^, a total of 56 signs of Indian pangolin were recorded within the research area. Amongst the 56 signs, 46 were burrows (living burrows (53.57%) and feeding burrows (28.57%)). Digging was observed nine (16.07%) times, along with one direct sighting (1.7%). Our results revealed a population estimate of only 29 pangolins in the Nowshera district, with a population density of 0.013 individuals/km^2^. Later, MaxEnt was applied to the species’ presence points, along with climatic and topographical variables. The MaxEnt model accuracy was good (AUC = 0.811). Of the total area studied, 210 km^2^ (12.01%) were highly suitable and 238 km^2^ (13.61%) were moderately suitable habitat for the Indian pangolin. To safeguard the fragile population and habitat of the Indian pangolin, we highly suggest strengthening watch and ward and law enforcement in the study area. By adopting a comprehensive approach that addresses both the direct threats to Indian pangolins and the underlying factors driving their decline, we can effectively protect this endangered species and ensure the preservation of its essential habitats for robust conservation.

## 1. Introduction

Population monitoring is one of the basic tenets of wildlife conservation, and is essential for improving knowledge of population dynamics and wildlife management [1,2]. Since many species’ population sizes are crucial markers for conservation and central to wildlife management goals, wildlife managers often assess management procedures by measuring these population sizes. Many species’ population sizes can serve as crucial markers for their conservation [3,4,5]. Conservation biologists investigate population sizes and trends by estimating the abundance of any target species [6,7]. Understanding and assessment of global or national level species extinction risks heavily depends on reliable population estimates [8]. Therefore, it is thought that one of the main concerns in conservation biology is to look into accurate population sizes, particularly for species that appear to be at risk of extinction or in danger of collapsing [9].

For propagation and completing life cycles, species require suitable surroundings called “habitats” [10]. Habitats are critical to a species’ survival because they provide the resources needed for it to persist [11,12]. Wild species tends to select the best habitats amongst the available habitats by complex behavioral processes, which ensure the fitness and survival of their populations [13]. However, natural wild habitats have been largely damaged by human activities, and this has put many species in danger of going extinct. Because of the terrible consequences of human activities, researchers are now trying to protect endangered species by maintaining their natural habitats [14]. Therefore, habitat research is vital in protecting and managing wildlife, as it offers a scientific justification for strengthening conservation laws [15,16].

It is possible to look at how wildlife interacts with its ecosystem and find possible habitats for endangered species by using ecological models like habitat suitability models (HSMs) [17]. HSMs aids in assessing a particular environment and identifies suitable substitute habitats for conservation-related initiatives [18]. The use of HSMs to map possible habitats for numerous species is growing [19]. The distribution, abundance, and habitat selection of species are all influenced by a number of anthropogenic and environmental factors, which ultimately affect species survival [20]. HSMs integrate data on species occurrence with climatic and other characteristics to estimate the environmental appropriateness of a species over a particular spatial range [21,22].

Small and medium-sized mammals play a variety of important roles in ecosystems, including soil aeration and seed and fungal dispersal [23,24]. They eat insects and other invertebrates and act as simultaneous food for several predators, preserving the diversity, distribution, and population dynamics of other animal species [25,26]. Several small and medium-sized mammal species have experienced sharp population declines due to human activities and environmental shifts, rendering them endangered [27,28], and the Indian pangolin is one of them [29].

The Indian pangolin is one of the four pangolin species found in Asia [30]. It is primarily myrmecophagous—having remarkable anatomical and behavioral characteristics to eat termites and ants [11]. The Indian pangolin, the only species of pangolin found in Pakistan, is arguably the least studied of all the Asian pangolin species [31]. Throughout its geographic range, the Indian pangolin lives in a variety of habitats, such as plains, farmlands, plateaus, and woods [30,32]. It occurs throughout Pakistan, with exception in the northern parts of Giglit-Baltistan (GB) and Khyber Pakhtunkhwa province [33].

The largest threat to this species is the illicit trade in its body scales, which are shipped to China and Vietnam from other range countries, where they are utilized in traditional remedies [34]. Due to the threat mentioned above, the Indian pangolin is also listed in Convention on International Trade in Endangered Species of Wild Fauna and Flora (CITES Appendix-I) [34]. Given the conservation status of the Indian pangolin, it is crucial to regularly assess its population across its entire range and identify priority landscapes for conservation. Therefore, the current study was designed with the aims to (1) investigate the population status of the Indian pangolin and the carrying capacity of the study area, and (2) to identify its suitable habitats in the Nowshera district of Khyber Pakhtunkhwa (KP) Province.

## 2. Materials and Methods

### 2.1. Study Area

The current study was carried out in Khyber Pakhtunkhwa Province’s Nowshera district, which is located at 34°005500 N and 71°5802900 E. The area consists of lowlands, hills, barren ground, agricultural land, urbanized townships, shrublands, and grasslands. This district is 1748 km^2^ in size and has an undulating terrain, with elevations between 234 and 1534 m above sea level (a.s.l). The district is divided into 47 union councils (UCs) for administrative purposes; Nizampur is the largest UC—now declared a sub-division—and Pabbi is the smallest. The average annual temperature of 24.4 °C and the average annual rainfall of 532 mm characterize the semiarid climate of our study area. The winter temperature falls below 0 °C in areas with high elevations (1534 m a.s.l), and it sporadically receives moderate snow. This region is dominated by scrub forests, which provide refuge for over 40 birds and 21 mammal species [35,36]. The Nowshera district comprises protected areas (PAs) i.e., game reserves and wildlife parks, as designated by the government [35,37].

### 2.2. Study Methods

#### 2.2.1. Sign Survey

A sign survey was carried out between July–September 2022 and November 2022–January 2023. Before the survey commencement, we performed a reconnaissance survey in the area along with wildlife department staff to mark potential habitats. We broadly divided the study area into two zones, namely, zone 1 and zone 2. Zone 1 included UCs of Dak Ismail Khel, Jaroba, Chapri, Spinkhak, Jallozai, Saleh Khana, Shahkot, Jabba, Bakhtai, and some portions of Azakhel. Zone 2 encompassed Nizampur sub-division, including Nizampur National Park (NNP). Later on, the aforementioned zones were divided into 5 × 5 km^2^ grids. To differentiate pangolin burrows and related signs from other species, we employed a mixed approach that combined local knowledge with verifiable evidence to identify pangolin burrows. Before starting the transect surveys, we interviewed wildlife rangers to identify the burrowing ranges of Indian pangolins and gathered local knowledge on distinguishing pangolin burrows from those of other animals in the forest and surrounding habitats. We learned that footprints, claw marks, fecal samples, scratch marks, and the shape of the burrow entrance were key indicators for accurately identifying pangolin burrows [30]. In order to confirm the species presence within each surveyed grid, we selected 10 random sampling points with a radius not less than 100 m. Sampling points were thoroughly searched for species signs, including burrows (living or feeding), digging (presumably digging referred to any non-burrow-related excavation by pangolins), scats, claw marks, or any direct sighting. A global positioning system (GPS) (Maverick v2.8) was used to record species presence locations [33,37,38].

#### 2.2.2. Population Estimation

To estimate the population size of the species, we employed the line transect method [39]. The length and width of each transect were approximately 4 km and 1 km, respectively. A total of 18 transects were established in zone 1 and 21 transects in zone 2. In order to apply the active burrow count method to determine population, transects were created in each of the selected sites [40]. The following formula was used for population density estimation [41].

D = n/A(1)

where “D” is population density, “n” is the number of active living burrows, and “A” is the total area sampled. To estimate total population of Indian pangolins in the Nowshera district, we extrapolated the density estimates of each zone over the whole district as follows:

N = D × total area of Nowshera district(2)

where “N” is the total pangolin population and “D” is the estimated population density.

Since pangolins are solitary animals, active burrows were assumed to have one individual. For estimating the carrying capacity of the area, we used the home range size of pangolins as reported by Gray et al. [42] (1 pangolin/1.58 km^2^) in relation to the highly suitable areas.

### 2.3. Habitat Suitability Modeling

To predict suitable habitats for Indian pangolin in the study area, we analyzed and processed the species presence data (obtained in the sign survey) by using maximum entropy modeling (MaxEnt) 3.3.3k [43]. MaxEnt’s precise simulation prediction skills and additional descriptive qualities make it a popular choice for habitat suitability modeling [10,44]. MaxEnt’s predictive accuracy has allowed it to outperform other species distribution models, even with presence-only and small datasets [37,38].

#### 2.3.1. Selecting Environmental Variables and Presence Data

Each environmental layer was converted to the same size and resolution, i.e., 1 × 1 km. ArcGIS 10.8 was used to convert Indian pangolin presence points into a vector file. In order to eliminate spatially correlated data points (those located within two kilometers), Indian pangolin presence records were checked for spatial autocorrelation using average nearest neighbor analysis in ArcGIS 10.8 (SDM toolkit) [42] and to guarantee individuality [45]. In the research area, 28 different locations generated habitat suitability models for Indian pangolins after spatial autocorrelation. A set of 26 environmental variables were first taken into consideration (Table S1). Nineteen of the 26 variables were bioclimatic, obtained from the WorldClim database (http://worldclim.org/, accessed on 3 September 2023), a collection of global climate layers collected from more than 4000 weather stations and including information on seasonality, extreme or limiting temperatures, precipitation, and annual averages that may have an impact on the distribution of species [46]. There were five topographic variables: soil, land cover, rivers, slope, and elevation. One variable human population density was anthropogenic. The Normalized Difference Vegetation Index (NDVI) was the vegetation-related variable. The topographic and bioclimatic variables had a spatial resolution of one kilometer. The bilinear resampling method was used to convert the resolution of the other variables to 1 km in 10.8 ArcGIS [38,47]. To eliminate strongly linked variables from the analysis, we used the Pearson correlation matrix in the R program (version 3.6.2) [48]. Ten non-correlated variables (r < 0.7) were kept after this procedure [37,38,47].

#### 2.3.2. Model Simulation and Evaluation

Data on the presence of Indian pangolins and a few other variables were changed to fit the MaxEnt software’s format (v 3.3.3k) [43]. Five repetitions were run using the typeset as a sub-sample, and we retained 5% of the data for random tests. We also utilized a random seed option. The other parameters, which included a regularization multiplier of one, a maximum of 10,000 randomly generated background points, and 500 maximum iterations with a 0.00001 convergence threshold, were left at their default values. The significance and contribution of each variable were ascertained using a jackknife estimator [49]. Sensitivity analysis was performed for each variable with a logistic output format. For every variable, sensitivity analysis was carried out using a logistic output format. Receiver operating characteristic (ROC) values were used to confirm the MaxEnt model’s success (Monterroso et al., 2009; Bai et al., 2018): 0.5–0.6 for rejected, 0.6–0.7 for poor, 0.7–0.8 for average, 0.8–0.9 for good, and 0.9–1.0 for exceptional ROC values [44,50]. The distribution of the appropriate habitat for Indian pangolins was reclassified using the output results. In order to create a habitat suitability map, the ASCII output format file was loaded into ArcGIS 10.8 and converted into raster data. Reclassification of raster data was used to determine the suitable area [37,38].

## 3. Results

### 3.1. Species Presence Records

In the current study, a total of 56 signs of Indian pangolins were observed in the study area. Amongst the 56 signs, 46 were burrows (living burrows (53.57%) and feeding burrows (28.57%)). Digging was observed nine (16.07%) times, along with one direct sighting (1.7%).

### 3.2. Estimated Population

A total of 39 transects were walked in both zones, yielding a sampling effort of 156 km^2^. The single direct sighting during the transect survey was counted as an active living burrow for population estimation. Results obtained in the current study revealed an estimated population of 23 individuals in the Nowshera district, with a population density of 0.013 individuals per km^2^ (Table 1).

### 3.3. Habitat Suitability

#### 3.3.1. MaxEnt Prediction Evaluation

**Figure 1:**
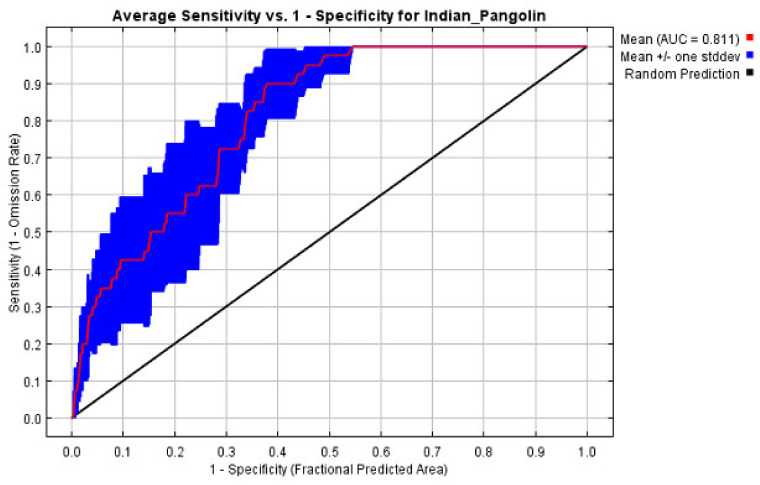
The ROC verification of distribution of suitable Indian pangolin habitat in the current study area.

Twenty-eight sites were utilized to create the current species distribution model (SDM) of the Indian pangolin in the research area after the spatially correlated locations were excluded. Based on the values of the area under the curve, our study produced a reliable and practical model (AUC). With an average AUC value of 0.811, the ROC findings (Figure 1) demonstrated the quality of the MaxEnt model’s predictions.

#### 3.3.2. Influential Factors Determining Habitat Suitability

**Figure 2:**
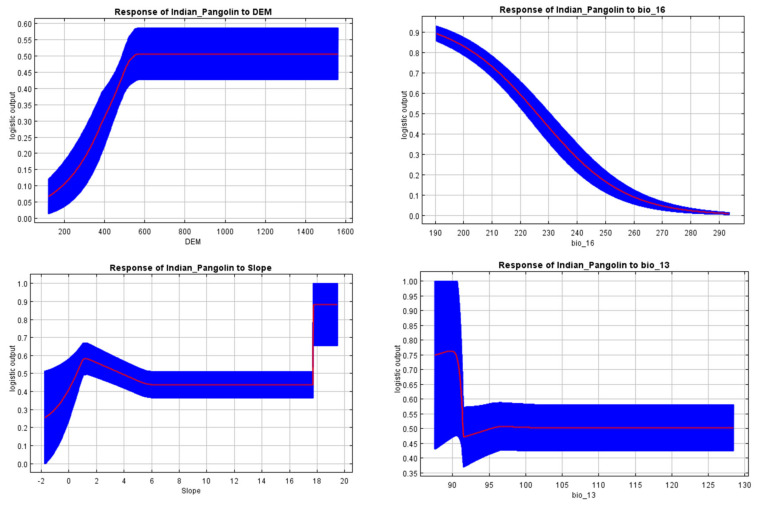
Response curves of the predictors for the presence of Indian pangolins in the research region. Note: The blue curves (two shades for categorical variables) reflect the mean +/− one standard deviation, whereas the red curves show the mean response of the five replicate MaxEnt runs. The Y-axis displays the predicted habitat suitability value (logistic output), and the X-axis displays the range of the environmental predictors.

The contribution of each model to the current SDM of the Indian pangolin was determined by MaxEnt (Table 2, Figure 2). Results reveal that the digital elevation model (DEM) contributed 45.5% to the habitat selection of Indian pangolins. Other variables with maximum contribution in habitat selection of Indian pangolins were bio16 (17.7%), slope (14.3%), and bio13 (7%). The variables with the lowest contribution rates were bio8 (1.3%) and bio 14 (0.7%).

**Figure 3:**
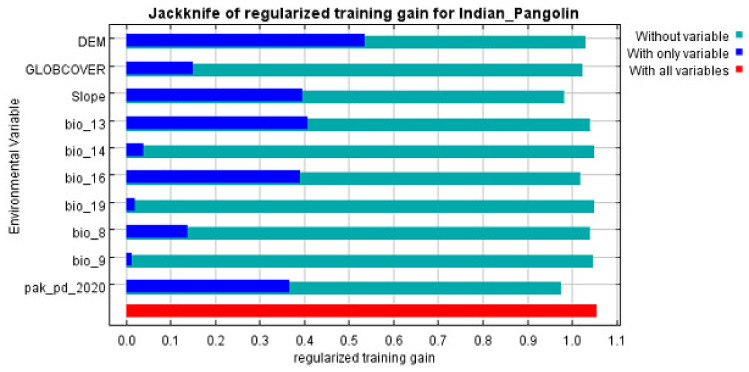
The Indian pangolin habitat suitability model’s regularized training gain of the variables tested.

The jackknife test results show that the DEM provided the most valuable information on its own, yielding the highest gain when used independently. Conversely, when pak_pd_2020 was removed, the gain dropped the most, indicating that this variable contained unique data not captured by the others. The displayed values represent averages across multiple runs (Figure 3).

#### 3.3.3. Distribution of Indian Pangolin Suitable Habitats

The habitat suitability map produced through MaxEnt modeling showed that highly suitable habitats of Indian pangolins are present in the southern and southwestern parts of the study area. These southwestern patches of highly suitable areas are located in the buffer zones of Cherat Wildlife Park. The southern patches exclusively fall within Nizampur National Park (NNP). Most of the moderately suitable habitats of Indian pangolins are located in and around protected areas encompassing southern, southeastern, and some central parts of the district. In addition, small patches of moderately suitable habitats were also identified in the western and northwestern sides towards Peshawar and Charsada districts, respectively. The districts mentioned above lie within the IUCN-declared range for the Indian pangolin. Unsuitable habitats were mostly restricted to western, northern, and eastern parts of the study area, which are the most heavily human-dominated areas, including city infrastructure, roads, and highways, along with railway networks and so on.

#### 3.3.4. Numerical Classification of Suitable Habitats

Based on the thresholds, the habitat suitability maps were divided into three groups: places that were highly suitable (0.46–0.92), moderately suitable (0.20–0.45), and unsuitable (0.00–0.19). The reclassified map was processed, and the results reveal that the study area’s inappropriate habitats made up 1299.9 km^2^ (74.36%), moderately suitable habitats 238 km^2^ (13.61%), and highly suitable habitats 210 km^2^ (12.01%).

#### 3.3.5. Carrying Capacity of the Study Area

Based on pangolin home range size, our results reveal a carrying capacity of 132 pangolins for the highly suitable areas in the Nowshera district.

## 4. Discussion

There is a paucity of information on the population dynamics of the pangolin species throughout its four range countries [41]. As far as we are aware, this is the first comprehensive attempt to determine the appropriate habitats and population status of Indian pangolins in this region. Keeping in view the estimated carrying capacity (132 pangolins), results obtained in this study reveal alarmingly low numbers (n = 23) of Indian pangolins, with a population density of 0.013 individuals/km^2^ in the study area. Other studies conducted in different parts of Pakistan reported comparatively higher population densities of the Indian pangolin. A recent study conducted in the Kohat district of KP province revealed a population density of 0.29 pangolins/km^2^ [41]. Extrapolating these results to the whole Kohat district yielded population estimates many folds higher (n = 867) compared to our findings. In the Chakwal district, which lies in the southern zone of Potohar Plateau, a population density of 1.0 individuals/km^2^ was reported [31]. A population density of 0.36 individuals/km^2^ was reported from Margalla Hills National Park, Islamabad [51]. Similarly, a population density of 0.77 individuals/km^2^ was reported from Azad Jammu and Kashmir (in and around Pir Lasura National Park) [52]. From the Mansehra district in KP, a population density of 0.28 individuals/km^2^ was reported [53].

Likewise, another recent study conducted in the adjacent Mardan district reported a population density of 0.09 individuals/km^2^, with an estimated population of 147 pangolins throughout the district [34]. On the other hand a very low population (n = 29) was reported for the southern belt (Bannu, Lakki Marwat, Tank, and Dera Ismail Khan districts) of KP province [54]. Compared to the findings of the aforementioned studies, we presume that the very low numbers of Indian pangolins in the Nowshera district and in the southern belt of the province can be attributed to several factors responsible for the low numbers, as described by Roberts [32] and Aisher [55]. In Pakistan, pangolins are being poached in several parts of the country, with the highest number of 275 pangolins in the Chakwal district between 2013–2018 [56]. According to the wildlife officials of the study area, several live pangolins and a potential quantity of scales has been seized in last few years from the study area (personal communication with Range Officer Wildlife, Nowshera Division). However, we believe that further in-depth research is needed to dig out the further causes, like prey base availability, etc., which, in association with poaching, could possibly contribute to such low numbers of Indian pangolins in these areas. Low population densities have been also reported from other parts of the country, as evident from the findings by Irshad et al. [40]. It is believed that low population densities indeed increase the risks of localized extinctions [55].

Our study revealed and highlighted the existing suitable habitats available for Indian pangolins in the Nowshera district. Our results show that a number of parameters affected habitat suitability, with the Digital Elevation Model having the greatest influence (45.5%), followed by bio16 (precipitation of wettest quarter) (17.7%) and slope (14.3%). Our study area elevation ranged between 200 and 1500 m a.s.l. Results suggest that Indian pangolin habitat suitability peaked at 200–500 m a.s.l, yet maintaining a constant probability across higher elevations. The Indian pangolin is mostly restricted in its distribution below 762 m a.s.l, suggesting a specific altitudinal range within which this species is predominantly found [32]. This altitude restriction may be due to various ecological factors, such as temperature, humidity, vegetation type, and prey availability. Yet in several Asian countries, the same species (*Manis crassicaudata*) can be found at elevations just lower than 1524 m [57]. Likewise, for other species of pangolin, i.e., the Chinese pangolin (*Manis pentadactyla*), it has been reported that their distribution is confined to areas where the elevation is either 1500 m a.s.l or just below [57]. In our study area, the most suitable habitats lay between 300 and 700 m a.s.l. However, we presume that in the Nowshera district, Indian pangolins may avoid higher elevations (900 m and above) because of the rocky terrain, which is hard to dig [34].

Our results also show that bio16 (17.7%) was the second most important factor. The response curve gradually decreased from 190 to 290 mm, indicating that the likelihood of occurrence or suitability increased with decreasing precipitation. The Indian pangolin is an insectivore that consumes termites and ants [11]. Indian pangolin prey species depend on wet environments to thrive and procreate. However, seasonal fluctuations and increases in precipitation seasonality decrease their numbers, which has an adverse effect on the pangolin [58,59].

Slopes less than 50° are preferred by pangolins [60]. Our results reveal an increasing trend along with the slope. At 6 degrees, the curve maintained a constant lateral line until it peaked at 18°. Findings by Shrestha et al. [61] and Khattak et al. [33] strongly validate our results. In the current study, we found that most of the suitable habitats lay in areas with slopes of 5–55°. Pangolins often favor mild slopes over vertical ones [62]. It is believed that the species’ avoidance of steep slopes may make it easier for them to move around and to limit high energy expenditures in daily excursions [33,63]. Our findings, however, go counter to those of Tamang et al. [64], who found that Chinese pangolins preferred much sharper slopes.

Our results show that the highly suitable habitats (210 km^2^) for the Indian pangolin are mostly located in and around buffer zones of the protected areas, including Nizampur National Park and Cherat Wildlife Park. Based on our findings, we presume that the available habitats for the Indian pangolin are possibly shrunken due to the rapid conversion of natural habitats into human settlements and infrastructures [65,66]. The highly suitable habitats in our study area harbor sub-tropical broad-leaved evergreen forests, along with several species of shrubs and tall grasses [35,67]. The Indian pangolin is highly adaptable to undulating terrains with sub-tropical thorn forests [32], which is a prominent geographical feature of the Nowshera district. The distribution of highly suitable habitats in areas with forests and thick vegetation revealed that the Indian pangolin prefers forests to agricultural lands or grasslands. Our findings are in agreement with those of Waseem et al. [65]. This preference suggests that pangolins may have access to more cover for shelter and protection compared to grasslands and agricultural areas [65].

## 5. Conclusions

Our research sheds light on the Indian pangolin population status and habitat appropriateness in the Nowshera district. According to the results of the current study, the Nowshera district still has a tiny, vulnerable population of Indian pangolins that require urgent care. Furthermore, the research region still has very favorable habitats. Nevertheless, given the current population condition, we strongly advise stepping up surveillance and monitoring procedures in the study area to save the ideal habitats that are still there. Every year, a close examination of the current population is necessary to evaluate population trends. Finally, we emphatically urge carrying out in-depth research to determine the fundamental causes—presumably poaching and food availability—possibly contributing to the low population of the Indian pangolin in the Nowshera district.

## Figures and Tables

**Table 1:** Estimated population density of Indian pangolin in the Nowshera district.

Site Name	Number of Living Burrows of Species		$\mathrm{Area}\;\mathrm{Sampled}\;(km^{2}$)	$D=N/A\;\mathrm{per}\;4\;km^{2}$	Total Number of Pangolins Extrapolated throughout the District
	Active Living Burrow	Inactive Living Burrow			
Zone 1	5	13	72	0.069	23
Zone 2	3	9	84	0.035	
Total	8	22	156	0.052 per 4 $km^{2}$	
Mean ± SE	4 ± 0.82			0.013 per $km^{2}$	

* *The total area of Nowshera District is 1748 km^2^*.

**Table 2:** Variables’ contribution to determining suitable habitats for Indian pangolins in the study area.

S.No.	Variable	Percent Contribution	Permutation Importance
1	DEM (Digital Elevation Model)	45.5	4.1
2	bio_16 (precipitation of wettest quarter)	17.7	21.9
3	Slope	14.3	4.4
4	bio_13 (precipitation of wettest month)	7	0.4
5	pak_pd_2020 (human population density)	5.4	52.7
6	bio_19 (precipitation of coldest quarter)	3.5	2.3
7	GLOBCOVER (land cover)	2.8	3.8
8	bio_9 (mean temprature of driest quarter)	1.8	4.4
9	bio_8 (mean temprature of wettest quarter)	1.3	4.5
10	bio_14 (precipitation of driest month)	0.7	1.6

## Data Availability

All the data obtained are presented in this article.

## Supplementary Materials

The following supporting information can be downloaded at: https://www.mdpi.com/article/10.3390/biology13090727/s1, Table S1: List of variables used in modeling potential habitats for Indian pangolin.
