# Supplementary material for: Insights into Population Status and Habitat Patches of Conservation Concern for the Endangered Indian Pangolin (Manis crassicaudata) in Nowshera District, Northwestern Pakistan
Source: Biology (Basel). 2024 Sep 16;13(9):727. doi: 10.3390/biology13090727 (PMC11428489; doi:10.3390/biology13090727)
Supplement: Supplementary file 1 [file biology-13-00727-s001.zip › biology-3176724-supplementary.pdf]

Table S1. List of variables used in modeling potential habitats for Indian pangolin.

Environmental variables	Unit	Source
annual mean temperature (bio_1))	Degrees Celsius	
mean diurnal range (mean of monthly [max temp—min temp]) (bio_2)	Degrees Celsius	
isothermality (Bio2/Bio7) (*100) (bio_3)	Percentage	
temperature seasonality (standard deviation *100) (bio_4)	Degrees Celsius	https://www.worldclim.org/data/worldclim21.html
maximum temperature of warmest month (bio_5)	Degrees Celsius	
minimum temperature of coldest month (bio_6)	Degrees Celsius	
temperature annual range (Bio_5-Bio_6) (bio_7)	Degrees Celsius	
mean temperature of wettest quarter (bio_8)	Degrees Celsius	
mean temperature of driest quarter (bio_9)	Degrees Celsius	
mean temperature of warmest quarter (bio_10)	Degrees Celsius	
mean temperature of coldest quarter (bio_11)	Degrees Celsius	
annual precipitation (bio_12)	Millimeters	
precipitation of wettest month (bio_13)	Millimeters	
precipitation of driest Month (bio_14)	Millimeters	https://www.worldclim.org/data/worldclim21.html
precipitation seasonality (coefficient of variation) (bio_15)	Fraction	
precipitation of wettest quarter (bio_16)	Millimeters	
precipitation of driest quarter (bio_17)	Millimeters	
precipitation of warmest quarter (bio_18)	Millimeters	
precipitation of coldest quarter (bio_19)	Millimeters	

human population density (pk_pd_2020)		WorldPop https://www.worldpop.org/doi/10.5258/SOTON/WP00674
global land cover 2009 (glc2009)		http://due.esrin.esa.int/page_globcover.php
elevation above sea level	Meter	NASA (SRTM)
slope of the area	Meter	created from SRTM 90m DEM
rivers		line Density tool in ArcGIS 10.8
digital soil map of the world		FAO, 2003
normalized difference vegetation index		USGS: http://edcsns17.cr.usgs.gov/glcc
